# Supplementary material for: Physcion-Matured Dendritic Cells Induce the Differentiation of Th1 Cells
Source: Int J Mol Sci. 2020 Mar 4;21(5):1753. doi: 10.3390/ijms21051753 (PMC7084398; doi:10.3390/ijms21051753)
Supplement: Supplementary file 1 [file ijms-21-01753-s001.pdf]

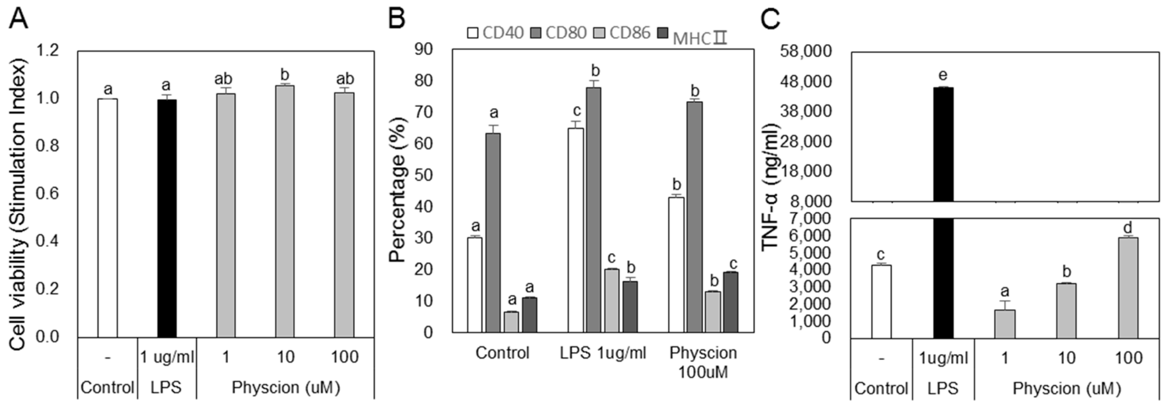

Supp. 1. Physcion increased surface molecule expressions and cytokines production in RAW264.7 macrophages. (A) Cell viability was assessed using CCK-8. (B) CD40, CD80, CD86, and MHCⅡ expressions in RAW 264.7 cells were assessed by FACS. (C) TNF-α levels in supernatants were determined by ELISA. Experiments were repeated twice. a, b, c, d, and e: different letters indicate significant differences ($p < 0.05$) as measured by one-way ANOVA with Duncan's multiple-range test.

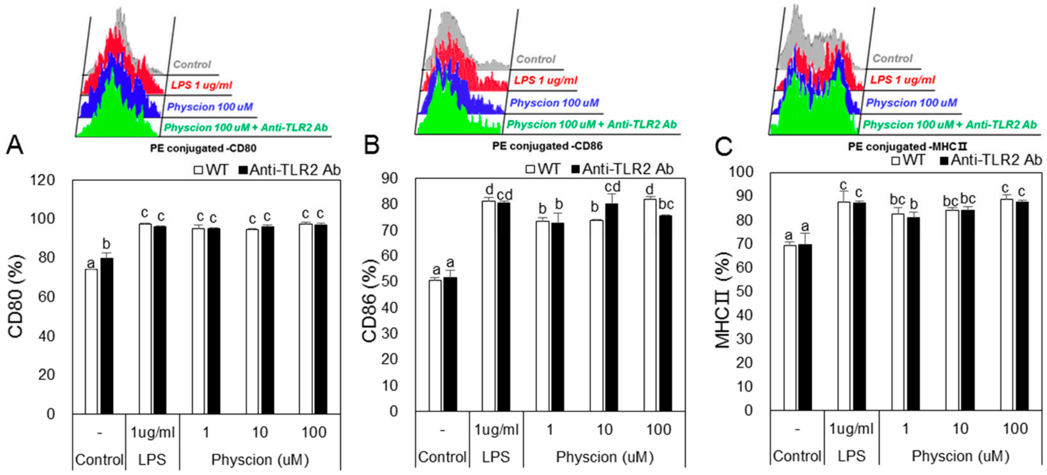

Supp. 2. Blocking TLR2 did not affect BMDC surface molecule expression. (A) Percentages of CD11c[+] CD80[+] cells, (B) CD11c[+]CD86[+] cells, (C) CD11c[+]MHCⅡ[+] cells from anti-TLR2 Ab treated or untreated BMDCs were measured by flow cytometry. Experiments were repeated twice. a, b, c, and d: different letters indicate significant differences ($p < 0.05$) as measured by one-way ANOVA with Duncan's multiple-range test.
